# Lower proportion of naïve peripheral CD8+ T cells and an unopposed pro-inflammatory response to human Cytomegalovirus proteins in vitro are associated with longer survival in very elderly people

**DOI:** 10.1007/s11357-012-9425-7

**Published:** 2012-06-04

**Authors:** Evelyna Derhovanessian, Andrea B. Maier, Karin Hähnel, Henning Zelba, Anton J. M. de Craen, Helene Roelofs, Eline P. Slagboom, Rudi G. J. Westendorp, Graham Pawelec

**Affiliations:** 1 Department of Internal Medicine II, Centre for Medical Research, University of Tübingen, Waldhörnlestrasse 22, 72072 Tübingen, Germany; 2 Department of Gerontology and Geriatrics, Leiden University Medical Centre, Albinusdreef 2, 2333 ZA Leiden, The Netherlands; 3 Netherlands Consortium for Healthy Aging, Leiden University Medical Center, Albinusdreef 2, 2333 ZA Leiden, The Netherlands; 4 Department of Immunohematology and Blood Transfusion, Leiden University Medical Centre, Albinusdreef 2, 2333 ZA Leiden, The Netherlands; 5 Department of Molecular Epidemiology, Leiden University Medical Centre, Einthovenweg 20, 2333 ZA Leiden, The Netherlands

**Keywords:** Immunosenescence, T-cell subsets, Mortality, CMV

## Abstract

The low percentages of naïve T cells commonly observed in elderly people are thought to be causally associated with mortality, primarily from infectious disease, and are taken as a hallmark of “immunosenescence”. Whether low levels of naive cells actually do associate with mortality has, however, not been tested in longitudinal studies. Here, we present correlations between peripheral T-cell phenotypes and 8-year survival in individuals from the population-based prospective Leiden 85-plus Study. Counter-intuitively, we found that a *lower* frequency of naïve CD8+ T cells (characterized as CD45RA+CCR7+CD27+CD28+) at baseline (>88 years) correlated with significantly *better* survival, while there was a tendency for the reciprocal accumulation of late-differentiated effector memory cells (CD45RA−CCR7−CD27−CD28−) also to associate with better survival. These findings suggest that better retention of memory cells specific for previously encountered antigens may provide a survival advantage in this particular population. Given the prevalence of Cytomegalovirus (CMV) and its reported association with immunosenescence, we tested whether memory for this potential pathogen was relevant to survival. We found that individuals mounting an exclusively pro-inflammatory ex vivo response (TNF, IFN-γ, IL-17) to the major CMV target molecules pp65 and IE1 had a significant survival advantage over those also having anti-inflammatory responses (IL-10). These findings suggest that higher levels of naïve T cells may not necessarily be associated with a survival advantage and imply that the nature of immunosurveillance against CMV may be crucial for remaining longevity, at least in the very elderly.

**Electronic supplementary material:**

The online version of this article (doi:10.1007/s11357-012-9425-7) contains supplementary material, which is available to authorized users.

## Introduction

Alterations to the cellular composition and function of the immune system occurring as individuals age are generally perceived as detrimental, and reflect a state commonly termed immunosenescence (Gruver et al. 2007). However, there is an alternative school of thought suggesting that the age-associated differences observed are adaptational, and indicate a remodeling of immunity depending on previous exposures and anticipated future exposures (Franceschi et al. 1995). A reduced frequency and number of peripheral naïve T cells and reciprocal accumulation of late-differentiated memory T cells are commonly accepted as hallmark characteristics of immunosenescence. These changes are believed to contribute to or even be responsible for the significantly higher incidence and severity of infectious disease as well as possibly cancer and autoimmune diseases and the decreased response to vaccination in the elderly (McElhaney and Effros 2009; Malaguarnera et al. 2010). Poor proliferative responses of T cells to mitogens have been associated with mortality in people of different age groups in several studies over the years (Murasko 1988; Wayne et al. 1990), suggesting a link between a simple measure of functional integrity of T-cell immunity and mortality. Due to the logistical constraints of investigating a long-lived species, most of the more sophisticated assessments of the differences in immunity between young and old have been based on cross-sectional studies, and so cannot formally demonstrate age-associated changes and their relationships to health measures.

One notable exception to this are the Swedish OCTO longitudinal studies performed over the last two decades in free-living individuals over 85 years of age. These investigations resulted in the emerging concept of an “immune risk profile” (IRP^2^), a cluster of slightly more sophisticated parameters, but also still including T-cell proliferation to mitogens, which was associated with 2-, 4-, and 6-year mortality on follow-up (Derhovanessian et al. 2009). In its original form, individuals in the IRP group (ca. 15 % of the population at baseline) were characterized by an inverted CD4/CD8 ratio (<1), increased absolute numbers of CD8+CD28− T cells per microliter of blood, poor mitogen-induced T-cell proliferative responses, low frequencies of B cells, and, initially surprisingly, seropositivity for Cytomegalovirus (CMV, the common persistent β-herpes virus HHV5) (Ferguson et al. 1995; Wikby et al. 1998; Olsson et al. 2000). We and others have shown that persistent infection with this virus is associated with accumulation of late-stage differentiated CD8+ T cells (Almanzar et al. 2005; Chidrawar et al. 2009; Derhovanessian et al. 2010). This has led to the increasingly accepted notion that CMV is one of or even the main driving force causing changes to immune signatures associated with immunosenescence (Pawelec and Derhovanessian 2011). Nonetheless, the longitudinal data from the OCTO studies came from people over 85 years of age selected for very good health at baseline, and although the presence of the IRP has been confirmed in the NONA studies of a representative population not in excellent health (Wikby et al. 2002), it was still limited to the inhabitants of one small southern Swedish city. Hence, its general applicability, even only to those over 85 years of age, has never been assessed.

In an effort to investigate this issue, here we have performed a detailed study using more sophisticated immunological analysis on a subgroup of individuals participating in the Leiden 85-plus Study, a prospective population-based cohort study of individuals at the age of 85 years living in Leiden, the Netherlands (van der Wiel et al. 2002). The cell samples studied here were from individuals 3 years after the original recruitment at baseline, i.e., at the age of 88 years, and with a prospective 8-year mortality data follow-up. Our findings reveal a set of immune parameters associating with 8-year mortality, but these are different from those identified for the IRP, and reveal an unexpected inverse relationship between the percentage of naive CD8+ T cells and survival. Thus, the widely accepted assumption that a more late-differentiated T-cell compartment, characterized by lower frequency of naïve cells and accumulated late-differentiated “senescent” cells, is always detrimental to survival may not necessarily be true, at least in the very elderly. Rather, as documented here, the presence of larger proportions of late-differentiated effector memory and effector cells, able to mount robust unopposed pro-inflammatory responses to major CMV antigens, is positively associated with longer survival in this population.

## Materials and methods

### Subjects

A subgroup of 50 participants of the Leiden 85-plus Study, a population-based, prospective study of inhabitants of Leiden, the Netherlands were analyzed here. The details of the Leiden 85-plus Study have been published previously (van der Wiel et al. 2002). Briefly, all inhabitants of Leiden born between 1912 and 1914 were invited to participate in the study between September 1997 and 1999. All participants were visited at their place of residence by medical staff and nurses. The Medical Ethics Committee of the Leiden University Medical Center approved the study, and informed consent was obtained from all participants. The blood samples analyzed in this study were taken in 2002. The characteristics of the 50 individuals studied here are listed in Table 1. All subjects were followed up for survival until January 2010 with a follow-up period of 8 years. Date and cause of death data were obtained from the Dutch civic registry.

**Table 1:** Characteristics of the subjects grouped according to CMV serostatus

	CMV−	CMV+
	*n* = 9	*n* = 41
Female, *n* (%)	5 (55.5)	33 (80.5)
White blood cells (×10^9^/l)	5.75 (1.3)	6.8 (1.8)
MMSE total score	27 (6.5)	25 (9.5)
GARS total score	46 (16.5)	37 (19.5)
GDS total score	4 (6.0)	1 (2.0)*

* Data from 31 individuals

Anti-CMV IgG titers were measured in plasma using a CMV IgG kit (ETI-CYTOK-G PLUS DiaSorin, Saluggia, Italy) based on enzyme immunoassay technology.

### Potential confounders

Global cognitive performance was assessed with the Mini-Mental State Examination (MMSE^3^) (Folstein et al. 1975). The 15-item Geriatric Depression Scale (GDS-15^4^) was used to measure depressive symptoms (Yesavage et al. 1982). Because of the limited validity of the GDS-15 in people with moderate and severe cognitive impairment, this was completed only by people with MMSE scores of more than 18. Ability to complete daily activities of living was measured by the Groningen Activity Restriction Scale (GARS) (Kempen et al. 1996). It assesses competence in abilities in nine personal basic activities of daily living (ADL) and nine instrumental activities of daily living (IADL). A summed score was calculated for basic IADL ranging from 9 (indicating ability to perform all activities without assistance or undue effort) to 36 (indicating disability). Other variables were gender and CMV serostatus.

### Flow cytometry analysis

After thawing, cryopreserved PBMCs were treated with human immunoglobulin, GAMUNEX (Bayer, Leverkusen, Germany), and ethidium monoazide (EMA) (Invitrogen, Karlsruhe, Germany) to block Fc receptors and label nonviable cells. Cells were then stained indirectly with anti-KLRG-1 primary antibody (Ab) (kindly provided by Prof. Hans-Peter Pircher, Freiburg, Germany) followed by staining with Pacific Orange-conjugated goat anti-mouse IgG (Invitrogen). After blocking with mouse serum (Chemicon/Millipore, Schwalbach, Germany), directly conjugated mAbs, CD3-PE (Calltag; Invitrogen), CD4-PerCP, CD8-allophycocyanin-Cy7, CCR7-PE-Cy7 (BD Biosciences, Heidelberg, Germany), CD27-allophycocyanin, CD45RA-Pacific Blue, CD28-Alexa Fluor 700 (BioLegend, San Diego, CA), and CD57-FITC (Immunotools, Freiburg, Germany) were added. After 20 min of incubation on ice, cells were washed and analyzed immediately on an LSR II cytometer with FACSDiva software (BD Biosciences). The spectral overlap between all channels was calculated automatically by the BD FACSDiva software, after measuring negative and single-color controls. Data were analyzed using FlowJo software (Tree Star, Portland, OR, USA). For data analysis, EMA-positive dead cells were excluded. In the viable gate, lymphocytes were gated in a forward light scatter versus side scatter dot blot according to their size and granularity. T cells within the lymphocyte gate were characterized as CD3+ cells. T-cell subsets were characterized according to surface expression of CD45RA, CCR7, CD27, and CD28 as described previously (Derhovanessian et al. 2010). Flow cytometry staining and data analysis were performed on blinded samples.

### Responsiveness to CMV antigens

PBMCs were thawed and allowed to rest for 8 h in X-Vivo15 medium (Cambrex) at 1 × 10^6^/ml at 37°C. pp65 and IE-1 PepMixes (JPT Technologies, Berlin, Germany) containing a mixture of short peptides covering the whole sequence of the molecules were used as antigens at a concentration of 1 μg/ml as recommended by the manufacturer. Peptides were added together with 1 μl/ml Golgi-Plug (BD Biosciences) to the cultures for 16 h. Medium alone and 50 ng/ml PMA (Sigma Aldrich, Munich, Germany) together with 750 ng/ml Ionomycin (Merck, Darmstadt, Germany) were used as negative and positive controls, respectively. The cells were then harvested, washed, and stained first indirectly for CD3 [OKT3 primary and Pacific orange-conjugated goat anti-mouse IgG secondary antibody (Invitrogen)]. After blocking with mouse serum (Chemicon/Millipore), the cells were stained with CD4-PerCP and CD8-APC-Cy7 (BD, Biosciences) mAbs. Following fixation and permeabilization using the BD Cytofix-Cytoperm solution (BD Biosciences), the cells were stained with IFNγ-PE-Cy7 (BD Biosciences), TNF-FITC (Miltenyi Biotec, Bergisch Gladbach, Germany), IL-17-PerCP-Cy5.5, and IL-10-Pacific Blue (eBioscience, Frankfurt, Germany). EMA was used to exclude dead cells. Samples were measured directly using the BD-LSR-II as described in the previous section. Cytokine secretion was only considered peptide specific when the amount of cytokine produced in response to a PepMix was at least twofold higher than the amount of cytokine produced in medium alone. These experiments could be performed in 21 CMV-seropositive individuals.

### Statistical analyses

Two independent groups were compared using the Mann–Whitney *U* test. Survival analysis was performed using the Cox regression analysis and adjustment of the hazard ratios (HR) of mortality for potential confounders, including gender, CMV serostatus, and markers of frailty (GARS, MMSE, and GDS). We applied Kaplan–Meier curves to display survival according to responsiveness to CMV antigens. Correlations between different T-cell subsets were analyzed using Spearman’s correlation analysis. All analyses were performed by Graph Pad Prism v3 or SPSS for Windows (SPSS Inc., Chicago, USA), version 17. *p* values <0.05 were considered significant.

## Results

### CMV and the distribution of T-cell subsets in the very elderly

**Fig. 1:**
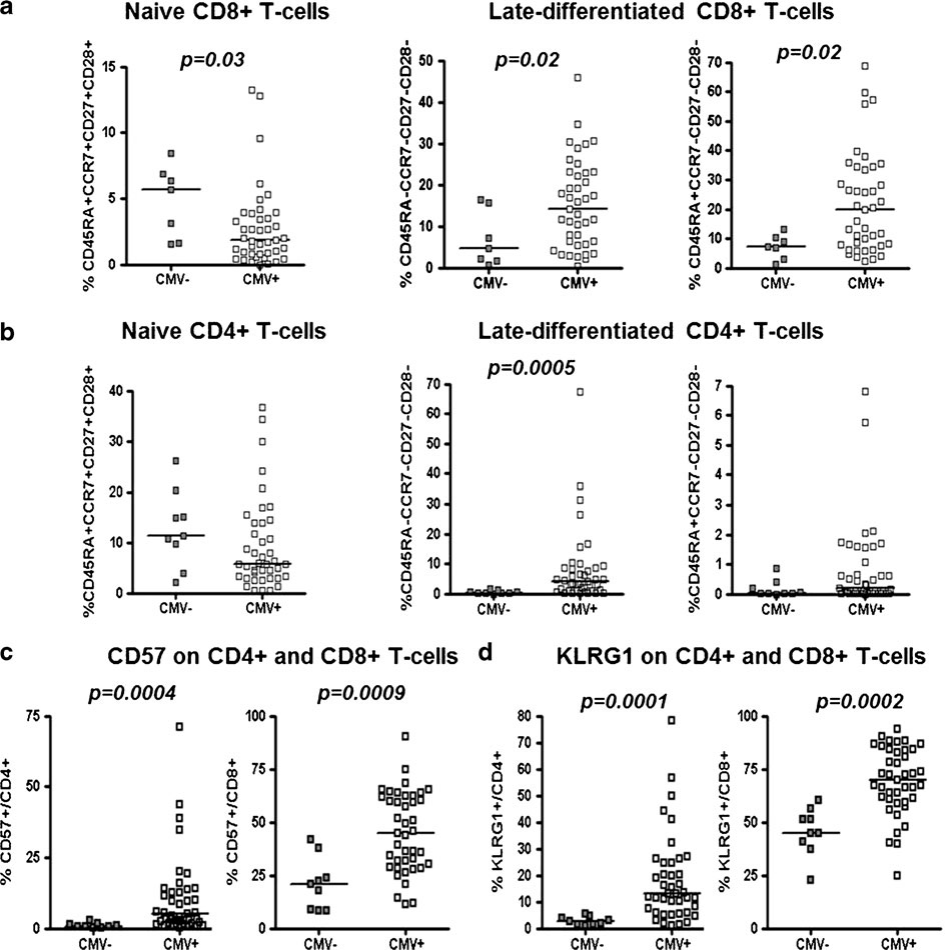
CMV seropositivity and distribution of T-cell phenotypes. Frequency of naïve and late-differentiated memory T cells within CD8+ (**a**) and CD4+ T cells (**b**) in seven CMV-seronegative (CMV−) and 41 CMV-seropositive (CMV+) individuals. Frequency of T cells carrying putative senescence markers CD57+ (**c**) and KLRG-1 (**d**) on CD4+ and CD8+ T cells were compared in the same individuals. *Horizontal bars* represent the median of each group

Considering its marked effect on the distribution of different T-cell subsets, particularly in the CD8 compartment and its inclusion as one of the parameters of the IRP, we first determined the impact of CMV infection in our cohort of individuals over the age of 88 years at baseline. Characteristics of the individuals grouped according to CMV serostatus are given in Table 1. Frequencies of different naïve and memory T-cell subsets were analyzed using 10-color flow cytometry, and different subsets were compared between CMV-seropositive and -seronegative individuals. As seen in other studies of different populations, we observed a strong association of CMV seropositivity with the distribution of different T-cell phenotypes; within the CD8 compartment, on average, there was a markedly lower percentage of naïve cells (N, defined as CD45RA+CCR7+CD27+CD28+, *p* = 0.03) and a reciprocal accumulation of late-differentiated effector memory (EM3^5^, CD45RA−CCR7−CD27−CD28−, *p* = 0.02) and late-differentiated effector (E^6^, CD45RA+CCR7−CD27−CD28−, *p* = 0.02) populations (Fig. 1a). Within CD4 T cells, there was a trend towards lower levels of naïve T cells in CMV-seropositive individuals, but this did not reach statistical significance (Fig. 1b, left-hand panel, *p* = 0.15). However, CMV infection was associated with accumulations of late-differentiated effector memory (EM3) T cells (*p* < 0.001), even up to 70 % of all peripheral CD4+ cells in one individual (Fig. 1b, middle panel). We also observed the emergence of a small population of late-differentiated CD4+ effector cells, re-expressing CD45RA in CMV-seropositive individuals, which was absent in most of the CMV-seronegative donors (Fig. 1b, right-hand panel). Additionally, we determined the T-cell differentiation status at the individual donor level by calculating the ratio of late-differentiated effector memory and effector subsets divided by the frequency of naïve T cells for each person. CMV-seronegative individuals had a median “differentiation index” of 0.03 (IQR 0.007–0.15) and 2.88 (IQR 0.7–9) for CD4+ and CD8+ T cells, respectively. In contrast, in CMV-seropositive individuals, the differentiation index for CD4+ (0.77, IQR 0.15–1.59) and CD8+ T cells (23.92, IQR 9.5–54.9) was significantly higher (*p* = 0.002 and *p* = 0.003, respectively). Consistent with this, there was a marked accumulation of CD4+ and CD8+ T cells carrying the putative very late differentiation or “senescence” markers CD57 and KLRG1 in CMV-seropositive individuals (Fig. 1c, d).

### Lower frequency of naïve CD8+ T cells is associated with better survival

Next we sought to determine if the large inter-individual variability seen for the T cell phenotypes, particularly in CMV-seropositive individuals, had any impact on 8-year survival in this very elderly cohort. Cox regression analysis was performed to calculate hazard ratios for the study population (Table 2). This revealed a significant *inverse* correlation between the percentage of naïve CD8+ T cells and survival, even after adjusting for gender, CMV serostatus, and frailty markers. Other T-cell phenotypes did not correlate with survival (Table 2). In order to further characterize the individuals with low naïve T-cell frequencies, a correlation analysis between this population and different memory cell phenotypes within both CD4+ and CD8+ T cells was performed. A low percentage of CD8+ naïve cells significantly correlated with a higher frequency of total CD8+ cells and the EM3 phenotype (Table 3). Although no direct correlation between naïve CD8+ T cells and the E phenotype was observed (*p* = 0.07, Table 3), individuals with lower frequency of naïve T cells did harbor higher frequency of both EM3 and E cells (Table 3).

**Table 2:** T-cell differentiation stages and survival

			HR	95 % CI	*p* values
CD4+ cells					
Naïve	Model 1	Gender-adjusted	1.02	0.98–1.06	0.26
	Model 2	model 1 + CMV	1.02	0.98–1.06	0.26
	Model 3	Model 2 + frailty	1.03	0.98–1.08	0.18
Late-differentiated effector memory	Model 1	Gender adjusted	1.01	0.99–1.04	0.4
	Model 2	Model 1 + CMV	1.01	0.98–1.04	0.36
	Model 3	Model 2 + frailty	1	0.98–1.04	0.57
Late-differentiated effector	Model 1	Gender adjusted	0.91	0.69–1.2	0.5
	Model 2	Model 1 + CMV	0.91	0.69–1.2	0.51
	Model 3	Model 2 + frailty	0.85	0.64–1.16	0.31
Differentiation index	Model 1	Gender adjusted	1	0.99–1.03	0.45
	Model 2	Model 1 + CMV	1	0.99–1.03	0.43
	Model 3	Model 2 + frailty	1	0.99–1.04	0.53
CD8+ cells					
Naïve	Model 1	Gender adjusted	1.14	1.02–1.28	0.025
	Model 2	Model 1 + CMV	1.16	1.03–1.3	0.015
	Model 3	Model 2 + frailty	1.17	1.02–1.33	0.023
Late-differentiated effector memory	Model 1	Gender adjusted	0.98	0.95–1.01	0.18
	Model 2	Model 1 + CMV	0.98	0.94–1.01	0.15
	Model 3	Model 2 + frailty	0.97	0.94–1.01	0.14
Late-differentiated effector	Model 1	Gender adjusted	1.01	0.99–1.03	0.47
	Model 2	Model 1 + CMV	1.01	0.99–1.03	0.49
	Model 3	Model 2 + frailty	1.01	0.99–1.03	0.61
Differentiation index	Model 1	Gender adjusted	1	0.99–1.0	0.54
	Model 2	Model 1 + CMV	1	0.99–1.0	0.53
	Model 3	Model 2 + frailty	1	0.99–1.0	0.79

Cox regression analysis adjusted for possible confounders

*HR* hazard ratio, *CI* confidence interval, *CMV* cytomegalovirus, *frailty* activities of daily living, Mini-Mental State Examination, Geriatric Depression Scale

**Table 3:** Correlation analysis between the frequency of CD8+ naïve cells (CD45RA+CCR7+CD27+CD28+) and different CD8 differentiation stages in 50 individuals

Parameter	Spearman *r*	95 % CI	*p* value
CD8+/CD3+	−0.3794	−0.6004 to −0.1045	0.006
CD45RA−CCR7−CD27−CD28−/CD8 (EM3)	−0.5084	−0.6937 to −0.2601	<0.001
CD45RA+CCR7−CD27−CD28−)/CD8 (E)	−0.2531	−0.5029 to 0.03565	0.076
EM3+E	−0.52	−0.7046 to −0.2799	<0.001

### *In vitro* pro-inflammatory response against CMV antigens in the absence of an anti-inflammatory response correlates with better survival

**Fig. 2:**
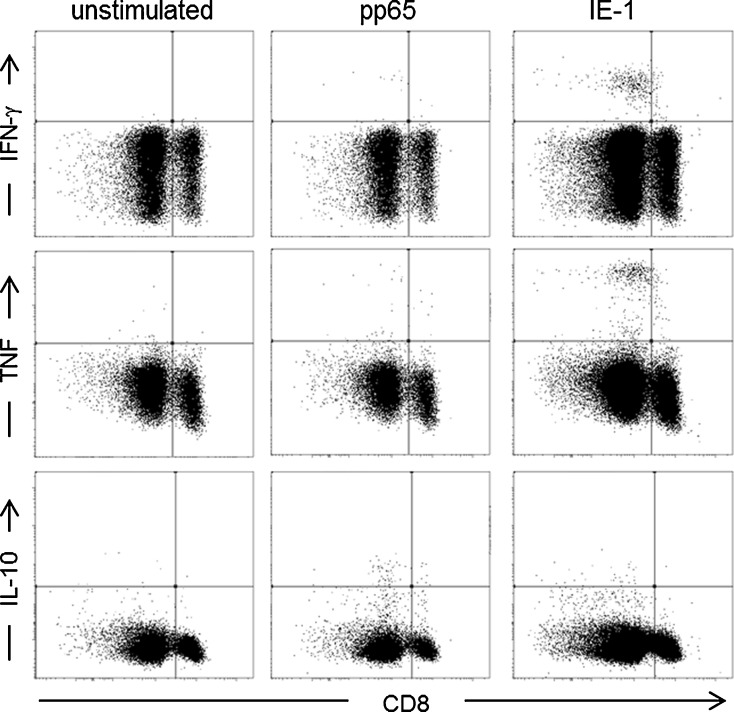
FACS plots demonstrating production of INFγ (*upper row*), TNF (*middle row*), and IL-10 (*lower row*) in the CD3+ gated population. The unstimulated control (shown in the *left-hand panel*) was used to set the cut-off for cytokine production

**Fig. 3:**
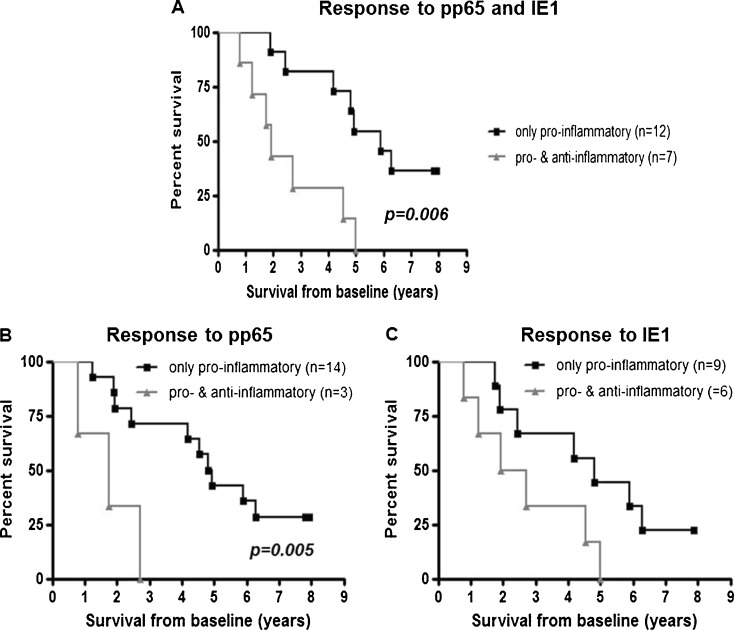
Correlation analysis between the *in vitro* immune response against immunodominant CMV antigens and mortality in individuals 88 years old at baseline. Kaplan–Meier survival curves for 21 CMV-seropositive individuals according to response to either pp65 or IE1 (**a**), or pp65 or IE1 (**b**) are shown. Donors were grouped according to the type of cytokine response; only pro-inflammatory—IFNγ, TNF, and/or IL-17 in the absence of any IL-10 production (*black line*), pro-/anti-inflammatory—IL-10 production by CD4+ and/or CD8+ T cells together or in the absence of pro-inflammatory cytokines (*gray line*)

Because subjects with lower percentages of naïve T cells possess higher levels of late-differentiated memory populations, we hypothesized that the survival advantage for individuals with few naïve cells might be correlated with a higher functional capacity for responses against previously encountered infectious agents. Because the majority of individuals in our study were CMV seropositive, we used CMV antigens as a model to assess this; we analyzed the *ex vivo* cytokine response of CMV-seropositive individuals against pools of overlapping peptides encompassing pp65 and IE1, two immunodominant CMV T-cell antigens. Responses against one or both antigens could be detected in 18/21 (85.7 %) CMV-seropositive individuals. In the majority of responding individuals (15/18), immune responses against both antigens could be detected. One individual responded only to IE1 and two only against pp65. The main response against both antigens was of a pro-inflammatory type (IFNγ and/or TNF); however, some individuals also responded with an anti-inflammatory cytokine profile in addition (IL-10 produced by either or both CD4+ and CD8+ T cells). Representative FACS plots of one donor with a strong pro-inflammatory response against IE-1 and IL-10 response against pp65 are shown in Fig. 2. Th17 responses against pp65 and IE1 were detected in eight and three individuals, respectively. Responders were grouped according to their cytokine profile: those with only pro-inflammatory responses (producing either or all of IFNγ, TNF, or IL-17) and those who also had an anti-inflammatory response (producing IL-10 as well) against any of the antigens. There was a significant survival disadvantage if an anti-inflammatory response against one or both of the antigens was induced in addition to the pro-inflammatory response (Fig. 3a). The difference remained significant after adjusting for gender and frailty markers (Table 4). Next, we sought to determine if an immune response against a certain antigen was associated with survival. For pp65, there was a significant survival advantage for individuals capable of mounting a pro-inflammatory response in the absence of an IL-10 response (Fig. 3b). A similar trend was observed for IE1, but this did not reach statistical significance (Fig. 3c). After adjusting for gender and frailty markers, we observed a significant correlation between the type of response mounted against both antigens and survival (Table 4).

**Table 4:** Mortality stratified by cytokine response (pro-inflammatory and pro- and anti-inflammatory) to pp65 and IE1 in CMV-seropositive donors (*n* = 18)

	Adjusted for gender			Adjusted for gender and frailty		
	HR	95 % CI	*p* value	HR	95 % CI	*p* value
Response to pp65 and IE1						
Pro-inflammatory response	0.11	0.03–0.48	0.003	0.1	0.02–0.055	0.007
Anti- and pro-inflammatory response	1 (ref)			1 (ref)		
Response to pp65						
Pro-inflammatory response	0.15	0.03–0.71	0.017	0.18	0.03–0.93	0.041
Anti- and pro–inflammatory response	1 (ref)			1 (ref)		
Response to IE1						
Pro-inflammatory response	0.15	0.03–0.69	0.015	0.08	0.01–0.72	0.024
Anti- and pro-inflammatory response	1 (ref)			1 (ref)		

*HR* hazard ratio, *CI* confidence interval, *frailty* activities of daily living, Mini-Mental State Examination, Geriatric Depression Scale

## Discussion

In this study, we present data demonstrating for the first time that the frequency of CD8+ naïve T cells (defined as CD45RA+CCR7+CD27+CD28+) at baseline *inversely* correlates with 8-year survival in individuals in the Leiden 85+ study over 88 years of age at the time of blood sampling, contrary to initial expectations. This was unanticipated because decreased levels of naïve T cells, one of the hallmark features of immunosenescence commonly observed in the elderly, has often been postulated to be the reason for increased mortality from infectious disease in this age group. Nonetheless, these data are consistent with earlier work from the Swedish OCTO/NONA longitudinal studies, in individuals over the age of 85 years, where the number of naïve T cells had not been part of a cluster of immune parameters correlating with mortality on 2-, 4-, and 6-year follow-up (Wikby et al. 2005). One interpretation of our findings is that a decreased level of naïve cells is reciprocated by accumulations of more differentiated effector cell types, which are cytotoxic and potent cytokine producers, and crucial for immunosurveillance against previously encountered infectious agents. Indeed, in the cohort studied here, the frequency of naïve CD8+ T cells correlated significantly with the frequency of late-differentiated memory T cells, which produce large amounts of granzyme, perforin, and IFNγ and have high cytotoxic activity, at least when tested in middle-aged individuals (Romero et al. 2007). Perhaps, in a modern society, sessile elderly individuals are more likely to be challenged by previously encountered rather than novel pathogens; thus, the higher frequency of effector memory-type cells would be more beneficial. Supporting this hypothesis, a strong pro-inflammatory response after challenge with CMV antigens *in vitro* in infected individuals in the absence of an opposing anti-inflammatory response was associated with better survival. The ability to mount a pro-inflammatory response in the absence of an anti-inflammatory response would also help protect against re-infection with any other pathogen, conferring a survival benefit. Similarly, in the larger cohort of the whole Leiden 85-plus Study, an unopposed pro-inflammatory response of the innate arm of the immune system *in vitro* was also associated with better survival (Wijsman et al. 2011). These data obtained from the individuals at the age of 85 and using different stimulation and read-out methods further strengthen our findings here.

Latent infection with CMV is present in the majority (ca. 85 %) of the elderly (but at 100 % in the IRP group) in different populations (Crough and Khanna 2009). Like the other viruses of the Herpes family, CMV can reactivate in immunocompromised, but also in critically ill immunocompetent, individuals, and causes severe disease and death (Cook et al. 1998; Jaber et al. 2005; Limaye et al. 2008). The strong impact of a latent infection with this virus on the distribution of the T-cell phenotypes of the host demonstrated here as well as in several other studies (Pawelec et al. 2009) reflects the challenge faced by the immune system in the presence of this latent infection. Clonal expansions of CD8+ T cells specific for CMV have been demonstrated in the elderly in numerous studies (Pawelec et al. 2009). Interestingly, the number of such clonal expansions was significantly lower in individuals in the IRP group compared with individuals in the non-IRP group in the terminal phase of life (Hadrup et al. 2006). Furthermore, a limited number of clonal expansions was shown to correlate with mortality in a small group of CMV-seropositive nonagenarians (Hadrup et al. 2006), suggesting that decreased immunological control of this virus might be associated with incipient mortality. Supporting this notion, in this study using an *in vitro* model of CMV reactivation, we have demonstrated that the nature of the anti-CMV response may indeed be associated with better survival. Ample evidence from transplant patients suggests a critical role for both pp65- and IE-1-specific pro-inflammatory T cells (secreting IFNγ) in controlling viral replication and protecting from CMV disease (Lacey et al. 2005; Crough et al. 2007; Ganepola et al. 2007; Nebbia et al. 2008). It is not yet clear if CMV reactivates more frequently in the elderly and what the clinical implications of a latent CMV infection are. In one study, CMV DNA could be detected more frequently in the urine of elderly subjects but not in the young (Stowe et al. 2007), suggesting some loss of control of the virus by the immune system in older people. Reactivation of other herpes viruses, like Epstein Barr Virus and Varicella Zoster Virus, also occurs in the elderly (Gilden et al. 2003; Stowe et al. 2007). Even if not directly leading to mortality, increased reactivation of CMV could render the individual more susceptible to infection with other opportunistic bacteria as well as contributing to increased low-level pro-inflammatory status, in itself pathologic in the elderly (Crough and Khanna 2009). Accordingly, three studies have demonstrated a significant correlation between anti-CMV antibody titers and all-cause as well as cardiovascular disease-related mortality (Strandberg et al. 2009; Roberts et al. 2010; Simanek et al. 2011). Considering the well-documented increased pro-inflammatory status in the elderly, partly caused by CMV, and the pathological effects attributed to it (Franceschi 2007; Sansoni et al. 2008), the survival benefit of mediating a pro-inflammatory-type response against CMV observed in our study might seem paradoxical. It is of note that six of the seven deaths within the pro-inflammatory responder group were due to cardiovascular disease or cancer compared to only three of seven deaths in individuals with an anti-inflammatory response. Although the number of individuals tested for cytokine response in our study is too low to draw any firm conclusions, our data suggest a fine line between the benefits of having a strong pro-inflammatory response, which would allow a better control of infections, and the negative side effects accompanying it in terms of inflammation-associated pathologies, such as cardiovascular diseases or cancer.

Numerous studies dating as far back as the 1980s have repeatedly shown that vaccination with influenza is associated with a strong reduction in not only pneumonia- or flu-related but surprisingly also all-cause- as well as cardiovascular mortality rates (Barker and Mullooly 1980; Nichol et al. 2003, 2007; Campitelli et al. 2010). In the cohort studied here, 60 % of the deaths occurred between November through March, the influenza season. If our findings modeled by CMV do apply to other memory antigens such as flu, this would translate to better protection from this disease as well, possibly explaining the observed survival advantage.

We also observed an inverse correlation between the frequency of naïve T cells and total CD8+ T cells. Thus, probably through compensatory homeostatic mechanisms, individuals with a lower frequency of naïve cells possess more CD8+ T cells and therefore a larger arsenal of highly functional EM3 cells. Interestingly, however, the mean frequency of CD8+ T cells in the study population (23.5 %) was similar to the non-IRP group of the OCTO study at baseline and the 2-year follow-up (Ferguson et al. 1995; Wikby et al. 1998). Thus, the expansion of CD8+ T cells was not to a detrimental degree and did not lead to an inverted CD4/CD8 ratio.

Apart from naïve CD8+ T cells, the frequency of other T-cell subsets did not have any impact on survival of the individuals in our study. This is in line with data from another study limited to women at a younger age (mean age of 77 years) and using fewer markers to characterize different T-cell subsets (Semba et al. 2005). Although in that work a lack of correlation between the frequency of naïve cells and mortality was reported, these cells were defined simply as CD45RA+. We now know that CD45RA expression alone is not sufficient to characterize truly naïve cells, especially within the CD8 compartment, as a late-differentiated memory subset re-expresses this isoform of the leukocyte common antigen phosphatase (Faint et al. 2001). High numbers of CD8+CD28− T cells, which was a parameter of the IRP identified in the OCTO/NONA studies, did not have any impact on 5-year survival in women over 75 years of age but was associated with frailty (Semba et al. 2005).

An inverted CD4/CD8 ratio, another parameter of the IRP, was observed only in one individual (2 %) in our study, unlike the OCTO and NONA studies, where 14 % of individuals over 85 years of age and 20 % of individuals older than 90 years had an inverted CD4/CD8 ratio (Ferguson et al. 1995; Wikby et al. 2002). The low frequency of individuals with a CD4/CD8 ratio <1 in our cohort might imply the natural selection of the fittest in the population. Indeed, in a representative group of the Dutch population at a younger age (70–81 years, *n* = 100) participating in the Leiden Longevity Study (data not shown), 20 % of subjects had a CD4/CD8 ratio <1. We also observed a lower 2-year mortality (16 %) in the individuals of the Leiden 85-plus Study compared with the OCTO study (26.5 %), indicating that the elderly studied here do indeed represent a healthier population, even though unlike the OCTO study, no exclusion criteria were applied. In fact, the mortality rate in the population excluded from the OCTO study was 54 % (Ferguson et al. 1995). These differences between the populations studied here, along with the different technical and statistical platforms applied, could explain the potential discrepancies observed between our data and those from the Swedish OCTO/NONA studies. In addition, medical advances over the intervening 15 years may have contributed to lower mortality in the elderly included in the Leiden 85-plus study.

Here, we have provided evidence that contrary to previously accepted models, a reduced frequency of naive CD8+ T cells, one of the hallmark features of immunosenescence, may not necessarily be detrimental for the elderly, at least those aged 88 years and older participating in the Leiden 85 plus Study. On the contrary, a lower frequency of naïve T cells, reciprocated by an accumulation of late-differentiated effector memory CD8 T cells, was associated with *better* survival. These unexpected findings point to the necessity of maintaining a delicate balance in the distribution of different T cells which would ensure a supply of sufficient effector CD8 cells to combat re-infection and/or maintain immunosurveillance against chronic infections, but not to a pathological level. Tipping this balance towards an inverted CD4/CD8 ratio, which seems to be irreversible in most individuals, can have detrimental consequences. This could happen for example through repeated reactivation of CMV due to loss of immunological control of the virus in CMV carriers. Our data demonstrating a direct correlation between the quality of the immune response to CMV antigens and mortality support this hypothesis.

Our findings, although obtained by necessity from a relatively small group of individuals, are nonetheless highly statistically significant and provide new insights into our understanding of the aging human immune system and its implications for survival and longevity, as well as eventual interventions to ensure appropriate functionality of immunity in old age.

## Electronic supplementary material

Below is the link to the electronic supplementary material.

ESM 1 (DOC 69 kb)

## Abbreviations

IRP: Immune risk profile
MMSE: Mini-Mental State Examination
GDS-15: 15-Item Geriatric Depression Scale
EM3: Effector memory 3
E: Effector
