# Supplementary material for: Lower proportion of naïve peripheral CD8+ T cells and an unopposed pro-inflammatory response to human Cytomegalovirus proteins in vitro are associated with longer survival in very elderly people
Source: Age (Dordr). 2012 Jun 4;35(4):1387–99. doi: 10.1007/s11357-012-9425-7 (PMC3705124; doi:10.1007/s11357-012-9425-7)
Supplement: Supplementary file 1 — (DOC 69 kb) [file 11357_2012_9425_MOESM1_ESM.doc]

**Supplemental Table 1.** Percentage of pp-65 and IE-1-specific T-cells from total CD4+ or CD8+ T-cells in 21 CMV-seropositive donors.

|  | **pp65** | | | | | | |  | | **IE1** | | | | | | | | |
| --- | --- | --- | --- | --- | --- | --- | --- | --- | --- | --- | --- | --- | --- | --- | --- | --- | --- | --- |
|  | **CD4** | | | |  | **CD8** | |  | **CD4** | | | | | |  | | **CD8** | |
|  | **IFNγ** | **TNF** | **IL-17** | **IL-10** |  | **IFNγ** | **TNF** |  | **IFFγ** | | **TNF** | **IL-17** | **IL-10** |  | | **IFNγ** | | **TNF** |
| **1** | 0.19 | 0.21 | 0 | 0 |  | 0.43 | 0.38 |  | 0 | | 0 | 0 | 0 |  | | 0 | | 0 |
| **2** | 0.26 | 0.40 | 0.12 | 0 |  | 0 | 0 |  | 0 | | 0.10 | 0 | 0 |  | | 0.21 | | 0.97 |
| **3** | 0.05 | 0.11 | 0 | 0 |  | 0.12 | 0.16 |  | 0.02 | | 0.04 | 0 | 0 |  | | 0 | | 0.13 |
| **4** | 0 | 0 | 0 | 0 |  | 0 | 0 |  | 0 | | 0 | 0 | 0.22 |  | | 0 | | 0 |
| **5** | 0.16 | 0.23 | 0 | 0 |  | 0.22 | 0.22 |  | 0 | | 0 | 0 | 0 |  | | 0 | | 0 |
| **6** | 0 | 0 | 0 | 0.32 |  | 0 | 0 |  | 0.18 | | 0 | 0 | 0.26 |  | | 0.71 | | 0.76 |
| **7** | 0 | 0 | 0 | 0 |  | 0 | 0 |  | 0 | | 0 | 0 | 0 |  | | 0 | | 0 |
| **8** | 1.64 | 1.68 | 0.55 | 0 |  | 1.40 | 1.33 |  | 0 | | 0 | 0 | 0.48 |  | | 0.54 | | 0.69 |
| **9** | 0.45 | 0.61 | 0 | 0.31 |  | 1.03 | 1.33 |  | 0.50 | | 0.52 | 0.28 | 0.50 |  | | 0.85 | | 1.59 |
| **10** | 0.37 | 0.39 | 0.06 | 0 |  | 0.10 | 0.09 |  | 0 | | 0 | 0 | 0 |  | | 0 | | 0 |
| **11** | 0 | 0 | 0 | 0 |  | 0 | 0 |  | 0 | | 0 | 0 | 0 |  | | 0 | | 0 |
| **12** | 0.13 | 0.21 | 0.04 | 0 |  | 0.39 | 0.51 |  | 0.12 | | 0.16 | 0 | 0 |  | | 0.41 | | 0.68 |
| **13** | 0.94 | 1.09 | 0.40 | 0 |  | 3.35 | 2.61 |  | 1.25 | | 1.32 | 0.47 | 0 |  | | 2.21 | | 3.06 |
| **14** | 0.53 | 0.57 | 0.12 | 0 |  | 0.10 | 0.18 |  | 3.00 | | 3.01 | 0.66 | 0.32 |  | | 1.52 | | 1.51 |
| **15** | 0 | 0.12 | 0 | 0.22 |  | 0 | 0.15 |  | 0.48 | | 0.39 | 0 | 0 |  | | 0.10 | | 0.15 |
| **16** | 0 | 0 | 0 | 0 |  | 0 | 0 |  | 0 | | 0 | 0 | 0 |  | | 0 | | 0 |
| **17** | 0.25 | 0.38 | 0.07 | 0 |  | 0.63 | 0.77 |  | 0 | | 0 | 0 | 0.13 |  | | 0.15 | | 0.13 |
| **18** | 0 | 0 | 0 | 0 |  | 0.27 | 0.31 |  | 0.02 | | 0 | 0 | 0 |  | | 0.40 | | 0.72 |
| **19** | 0.40 | 0.48 | 0 | 0 |  | 0.47 | 0.43 |  | 0 | | 0 | 0 | 0 |  | | 0.25 | | 0.37 |
| **20** | 0.50 | 0.60 | 0.20 | 0 |  | 0.97 | 0.96 |  | 0.87 | | 0.89 | 0 | 0 |  | | 3.17 | | 4.53 |
| **21** | 1.36 | 2.28 | 0.09 | 0 |  | 0.41 | 0.53 |  | 0 | | 0 | 0 | 0 |  | | 0.48 | | 0.74 |
